# Enzymatic C4‐Epimerization of UDP‐Glucuronic Acid: Precisely Steered Rotation of a Transient 4‐Keto Intermediate for an Inverted Reaction without Decarboxylation

**DOI:** 10.1002/anie.202211937

**Published:** 2022-12-15

**Authors:** Annika J. E. Borg, Oriol Esquivias, Joan Coines, Carme Rovira, Bernd Nidetzky

**Affiliations:** ^1^ Institute of Biotechnology and Biochemical Engineering Graz University of Technology Petersgasse 12/1 8010 Graz Austria; ^2^ Austrian Center of Industrial Biotechnology (acib) Krenngasse 37 8010 Graz Austria; ^3^ Department of Inorganic and Organic Chemistry (Section of Organic Chemistry) Institute of Computational and Theoretical Chemistry (IQTCUB) Martí i Franquès 1 08028 Barcelona Spain; ^4^ Institució Catalana de Recerca i Estudis Avançats (ICREA) Passeig Lluís Companys, 23 08010 Barcelona Spain; ^5^ Present address: Nostrum Biodiscovery Av. De Josep Tarradellas, 8–10 08029 Barcelona Spain

**Keywords:** Carbohydrates, Enzyme Catalysis, Epimerase, QM/MM, UDP-Glucuronic Acid

## Abstract

UDP‐glucuronic acid (UDP‐GlcA) 4‐epimerase illustrates an important problem regarding enzyme catalysis: balancing conformational flexibility with precise positioning. The enzyme coordinates the C4‐oxidation of the substrate by NAD^+^ and rotation of a decarboxylation‐prone β‐keto acid intermediate in the active site, enabling stereoinverting reduction of the keto group by NADH. We reveal the elusive rotational landscape of the 4‐keto intermediate. Distortion of the sugar ring into boat conformations induces torsional mobility in the enzyme's binding pocket. The rotational endpoints show that the 4‐keto sugar has an undistorted ^4^ *C*_1_ chair conformation. The equatorially placed carboxylate group disfavors decarboxylation of the 4‐keto sugar. Epimerase variants lead to decarboxylation upon removal of the binding interactions with the carboxylate group in the opposite rotational isomer of the substrate. Substitutions R185A/D convert the epimerase into UDP‐xylose synthases that decarboxylate UDP‐GlcA in stereospecific, configuration‐retaining reactions.

## Graphical abstract

Enzymatic C4‐epimerization of UDP‐glucuronic acid involves precisely steered rotation of a transient 4‐ketohexuronic acid intermediate that is facilitated by chair‐to‐boat distortion of a sugar ring. The enzyme thus retains stereoelectronic control and prevents even trace amounts of decarboxylation occurring. A link between conformational selection and epimerase or decarboxylase activity is suggested.

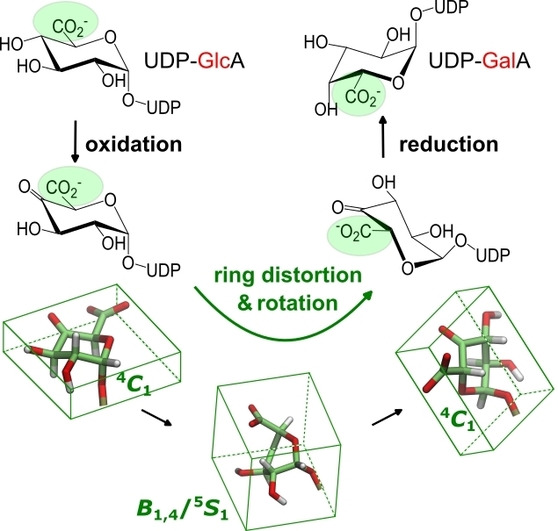

## Introduction

Enzymes catalyze chemical reactions with unrivaled efficiency.^[1]^ In the modern (i.e. dynamic) view of how enzymes operate, conformational sampling enabled by protein flexibility is fundamental to their specificity and efficiency.^[2]^ Not only is it crucial to coordinate the immediate catalytic event with other physical steps of the reaction, it is also key in the dynamic sampling of enzyme–substrate conformers that have electrostatics and internuclear distances tuned for the bond cleavage/formation.[^2^, ^3^] UDP‐d‐glucuronic acid (UDP‐GlcA) 4‐epimerase (UGAepi; EC 5.1.3.6) catalyzes stereoinversion at the C4‐position of UDP‐GlcA to provide d‐galacturonic acid for the synthesis of cell‐wall polysaccharides.[^4^, ^5^, ^6^, ^7^, ^8^, ^9^] UGAepi illustrates in many ways a general problem of fundamental importance in enzyme catalysis: in order to promote the reaction efficiently, the enzyme must achieve a fine balance between protein flexibility and precise substrate positioning.^[6]^

**Figure 1:**
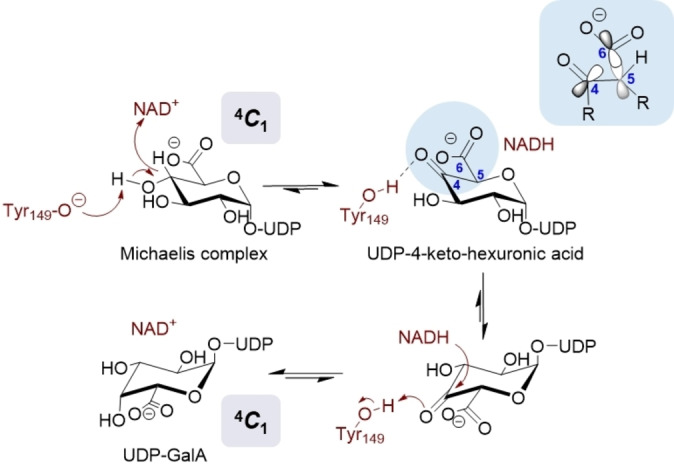
Proposed mechanism of UGAepi‐catalyzed interconversion of UDP‐GlcA and UDP‐GalA involving stereoelectronic control.^[6]^ Binding‐pocket interactions keeping the carboxylate group in an equatorial orientation, as in enzyme‐bound UDP‐GlcA and UDP‐GalA,^[5]^ would result in an orbital alignment that disfavors decarboxylation of the 4‐keto intermediate.

The UGAepi reaction consists of two catalytic steps in a canonical sugar nucleotide epimerase mechanism,[^3h^, ^10^, ^11^, ^12^, ^13^, ^14^, ^15^] such as that of UDP‐galactose 4‐epimerase (Figure 1): site‐specific C4‐oxidation of the substrate by a tightly bound NAD coenzyme; and non‐stereospecific reduction of a transient UDP‐4‐ketohexuronic acid intermediate by the enzyme‐NADH.[^12^, ^13^] To allow for hydrogen re‐addition to either face of the carbonyl group, the 4‐keto group must be accommodated flexibly within the enzyme binding pocket. It is generally believed that the 4‐keto sugar is able to rotate in the active site,[^4^, ^5^, ^6^, ^10^, ^11^, ^12^, ^13^, ^14^, ^15^] but how the enzyme enables torsional mobility of the bound intermediate is unknown and remains puzzling structurally.

The rotation step of the enzymatic mechanism receives additional significance from the fact that UGAepi has to counter decarboxylation of the metastable β‐keto acid species, as represented by its 4‐keto intermediate.[^4^, ^6^] It has been suggested that UGAepi exploits binding pocket interactions (revealed in enzyme complex structures with UDP‐GlcA and UDP‐GalA) to restrict the relative orientation of the intermediate's carboxylate and keto groups so that the decarboxylation is disfavored stereoelectronically.[^4^, ^5^, ^6^] Integrating conformational constraints for stereoelectronic control, however, poses a conundrum for the enzyme given the requirement for rotation of the 4‐keto intermediate. Herein, we report evidence from a combined computational and experimental analysis that delineates the full rotational landscape of the 4‐keto intermediate and shows its interconnection with the catalytic steps. Our results reveal the requirement for enzyme‐promoted distortion of the sugar ring to induce torsional mobility and to steer the rotation of the 4‐ketohexuronic acid moiety in the constrained UGAepi binding pocket. A role of conformational sampling linked to chemoselective catalysis by the epimerase is thus suggested. UGAepi variants designed to exhibit a major defect in the conformational sampling are discovered to act as primitive decarboxylases that promote the slow release of UDP‐xylose from UDP‐GlcA in completely stereospecific reactions. Overall, therefore, our study highlights with the example of UGAepi the significance of coordinated changes in conformation (coupled motions) for efficiency in multistep enzymatic catalysis.[^2a^, ^2f^, ^3^, ^6^]

## Results and Discussion

**Figure 2:**
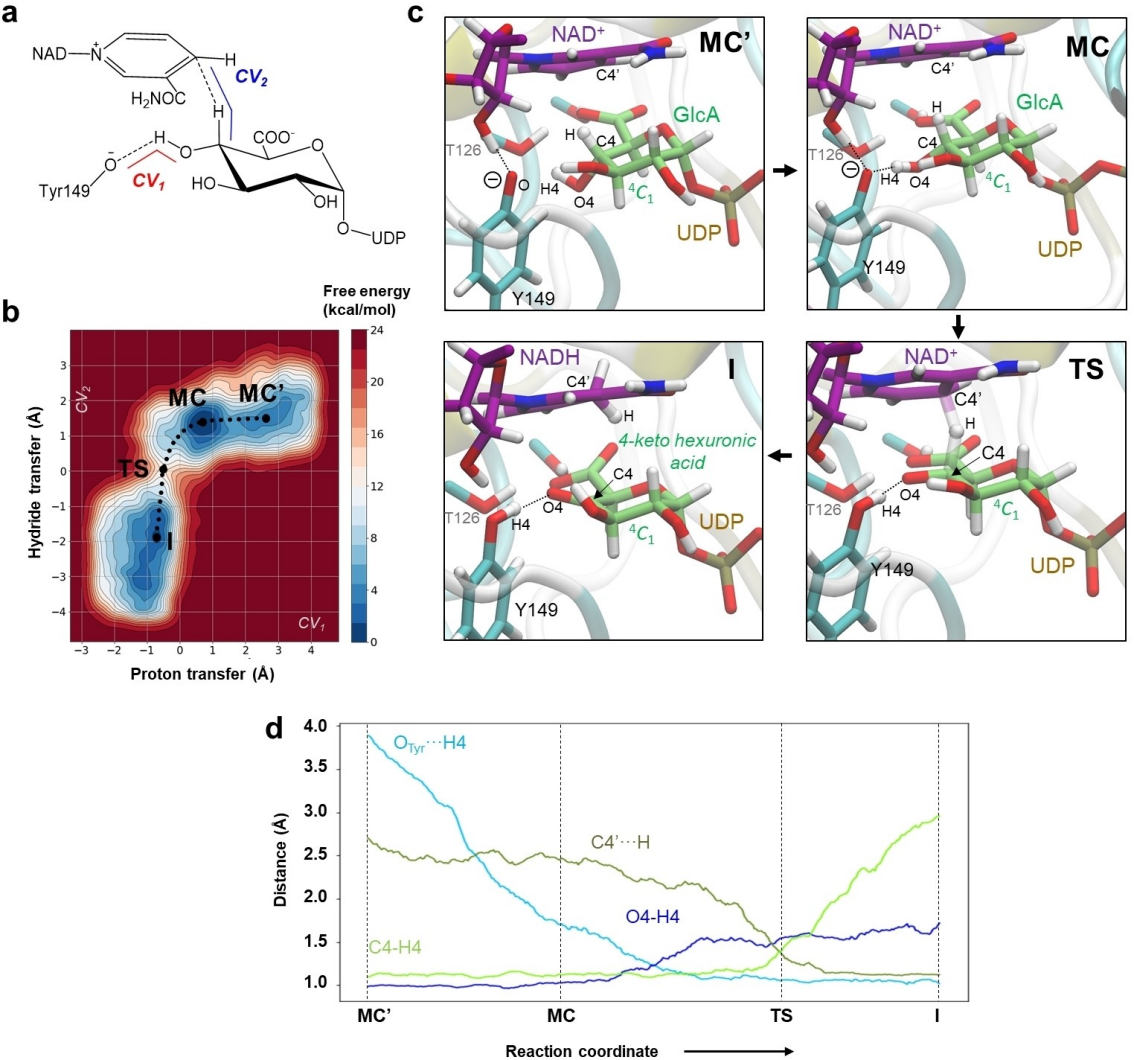
QM/MM metadynamics simulations of the oxidation of UDP‐GlcA catalyzed by BcUGAepi. a) Collective variables used. b) Free‐energy landscape (FEL) obtained from the simulation (isolines at 1 kcal mol^−1^). c) Representative structures of stationary states along the reaction coordinate. d) Evolution of the main catalytic distances along the minimum free energy pathway (reaction coordinate).

As a first step in our investigation, we analyzed the catalytic conversion of UDP‐GlcA into the 4‐keto intermediate with MD and QM/MM metadynamics methods (see the Supporting Information for methods). MD simulations (600 ns) starting from the UDP‐GlcA complex structure with BcUGAepi (enzyme from *Bacillus cereus*; PDB 6ZLD^[5]^) showed the pyranosyl moiety in a relaxed ^4^ *C*_1_ conformation throughout. The sugar hydrogen atom at C4 remains in proximity (2.6 Å distance on average) to the nicotinamide C4′ atom (Figure S1). Simultaneously, the 4‐OH group often engages in hydrogen bonding with the Y149 phenolate group (H4⋅⋅⋅O_Tyr_=1.9 Å, Figure S1). Y149 is highly conserved, and its proposed function is that of a general base for oxidation.^[4]^ With groups arranged plausibly for C−H bond cleavage under proton assistance, the observed conformations seem to be true representatives of the BcUGAepi Michaelis complex (Figure S1). One such conformation, corresponding to a snapshot of the dynamically equilibrated structure, was selected for detailed study of the catalytic process by QM/MM metadynamics. A large QM region (101 QM atoms; 1 082 223 MM atoms) was considered, including the GlcA unit, the phosphate groups of UDP, part of the NAD^+^, and the side chains of Y149 and T126. Two collective variables (CV) were defined to represent the proton (CV_1_) and hydride abstraction (CV_2_) of the overall oxidation (Figure 2a).

The results in Figure 2b,c show that the reaction effectively evolved from the proposed Michaelis complex (referred to as **MC**) towards the UDP‐4‐ketohexuronic acid/NADH complex (referred to as **I**, for intermediate). The two free‐energy minima assigned to **MC** and **I**, respectively, are practically isoenergetic (difference <1 kcal mol^−1^) and separated by a unique transition state (**TS**), indicative of a concerted reaction. The free‐energy barrier for the catalytic step (**MC**→**TS**) is 13.1 kcal mol^−1^. From transition‐state theory, this barrier corresponds to a rate constant of the immediate catalysis that is approximately 10^3^‐fold faster than the experimental *k*_cat_ (ca. 1 s^−1^; at 300 K). However, the observable *k*_cat_ value is not solely limited by the catalytic step. Kinetic isotope effects implicate a precatalytic rearrangement of the enzyme–substrate complex in the partial rate limitation of *k*_cat_.^[4]^ Crystal structures of UGAepi provide additional evidence in support of the “kinetic complexity” of the *k*_cat_ value, showing a conformational change of the protein (loop closure movement around the active site) associated with the UDP‐GlcA binding.^[5]^ It is important to note that our QM/MM metadynamics calculations start from the closed‐loop structure of the enzyme/UDP‐GlcA complex; therefore, such a conformational change was not modeled. Nonetheless, the calculations reveal that formation of the **MC** involves a transition from a secondary minimum (**MC′**), corresponding to enzyme conformers that have the Y149 phenolate hydrogen atom bonded to the ribosyl 2‐OH group of NAD^+^ (Figure 2c). Although the **MC′** conformers are nonproductive towards oxidation, their occurrence has mechanistic importance in two ways. First, flexible orientation of Y149, such as in **MC** and **MC′**, is thought to be important for proton relay in catalysis by the superfamily of enzymes (short‐chain dehydrogenases/reductases; SDRs) to which UGAepi belongs.^[16]^ Second, in addition to the energy barrier (**MC**→**TS**), the experimentally observed *k*_cat_ value includes the effect of nonproductive states. In other words, the computationally predicted *k*_cat_ value will be lowered by the fraction of the total enzyme present in the form of nonproductive conformers such as **MC′**, as well as the contribution of the prearrangement (substrate‐binding) step. We note that hydrogen–deuterium exchange could be an interesting technique in a future study to analyze the conformational changes associated with the immediate chemical event of catalysis by BcUGAepi.^[2c]^

The minimum free energy path of the catalytic oxidation (Figure 2b,d) reveals an asynchronous reaction, with the hydride transfer lagging far behind the proton transfer. The **MC** features a distinctly short hydrogen bond (Y149−O⋅⋅⋅H4=1.7 Å) that initiates the proton transfer (Table S1). On moving to the **TS**, the proton has been transferred completely, whereas the hydride transfer is about midway between the substrate and NAD^+^ carbon atoms (Figure 2d). As suggested by the shape of the free‐energy landscape (Figure 2b), it is primarily the hydride transfer that requires activation energy to proceed. With the exception of increased ring planarity arising from the presence of the 4‐keto group, as measured by the radial puckering coordinate (Figure S2), the hexuronic acid moiety remains in the ^4^ *C*_1_ conformation throughout.

**Figure 3:**
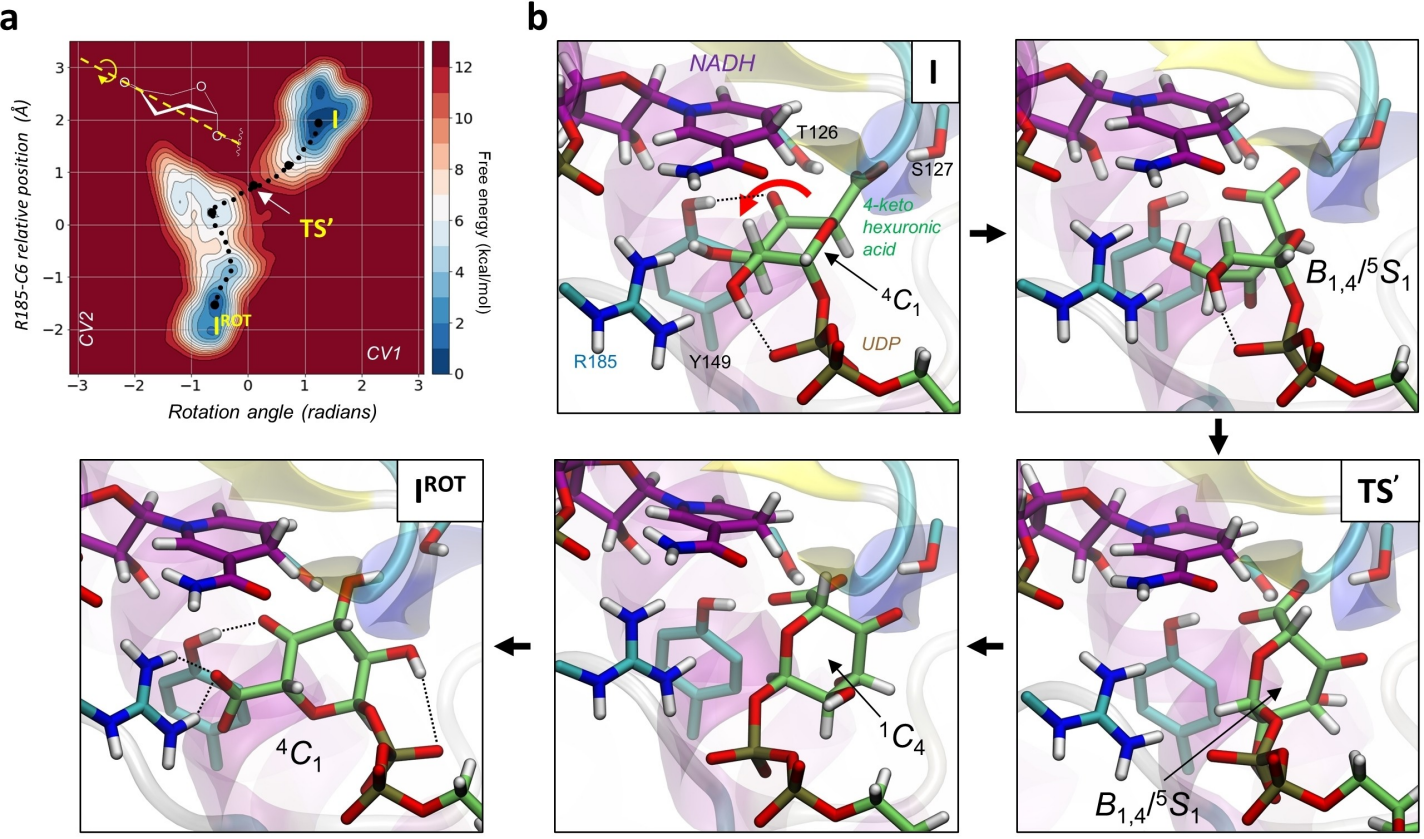
Rotational coordinate of the 4‐ketohexuronic acid in the active site of BcUGAepi obtained from metadynamics simulations. a) Free‐energy landscape (FEL) according to a representative metadynamics simulation of the reversible rotation. See Table S3 for the consistent reversibility of the rotation between **I** and **I^ROT^**. b) Representative structures of stationary and relevant states along the reaction coordinate. Full depiction of the structural changes in the rotational itinerary are shown in a Supporting Movie.

Starting from the emergent **I** conformer, we modeled the rotation of the 4‐keto intermediate. As no covalent bond is broken/formed in the process, force field‐based methods (classical MD with metadynamics) are suitable for the analysis. Given the lack of a force field to describe the 4‐ketohexuronic acid moiety, we developed one specifically (see the Supporting Information methods). The BcUGAepi complex structure with UDP‐GalA (PDB 6ZLL^[5]^) served as reference to assess the rotational endpoint conformers. Preliminary evidence indicated that a single CV (the torsion angle around the longitudinal axis of a ring) failed to populate enzyme conformers featuring full rotation of the pyranosyl ring, such that the carboxylate group would be placed on the opposite side to interact with R185. Adding a second CV to pull the carboxylate towards the R185 side chain successfully drove the rotational motion to completion (Figure S3). The two endpoints of the reversible rotation (**I**, **I^ROT^**) correspond to isoenergetic free energy minima on the conformational landscape. They are connected by a free energy barrier (**TS′**) of 10.8 kcal mol^−1^ (Figure 3a). Based on the difference in barrier height (+2.3 kcal mol^−1^), the rotation is expected to be about 60‐fold faster than the oxidation. This is consistent with previous biochemical evidence^[4]^ that shows that BcUGAepi‐NADH is below the detection limit during UDP‐GlcA epimerization under steady‐state conditions and requiring that both rotation and reduction with configurational inversion are fast compared to oxidation. Detailed analysis of the rotational coordinate (Figure 3b) reveals a complex coupled motion that departs substantially from a rigid‐body rotation. This motion involves the 4‐ketohexuronic acid moiety adopting a coordinated series of conformations to be accommodated within the enzyme binding pocket (see the Supporting Movie). Steered by a hydrogen bond from S127 and later Y149 to the sugar carboxylate group (Figure 3b), the pyranosyl ring initially distorts from ^4^ *C*_1_ to a high‐energy conformation that lies between *B*_1,4_ and ^5^ *S*_1_. The conformational rearrangement orients the carboxylate group axially, with the rotation‐promoting consequence that the accessible volume of the sugar is reduced and steric interactions with the dihydronicotinamide ring are minimized.

The catalytically relevant hydrogen bond from Y149 to the 4‐keto group (a characteristic feature of both **I** and **I^ROT^**) is broken during the rotation. Despite the extensive changes to the ring puckering involved in the rotation, the 4‐ketohexuronic acid retains the stabilizing interaction of its 2‐OH group with the β‐phosphate moiety of the UDP (Figure S4). The **TS′** state also features a distorted *B*_1,4_/^5^ *S*_1_ conformation. Simulation results suggest a ≥10 kcal mol^−1^ energetic benefit of rotation via **TS′** compared to simple rigid‐body rotation (obtained by “freezing” the sugar ring distortion, see Figure S5). After crossing the **TS′**, the pyranosyl moiety evolves towards an inverted chair (^1^ *C*_4_), keeping the carboxylate group in an axial orientation and hydrogen bonded to Y149 (Figure 3b). However, once the ring flip is completed, the sugar can relax into a ^4^ *C*_1_ chair conformation which positions the carboxylate group equatorially and so enables it to interact strongly with R185 (Figure 3b, **I^ROT^**). In both conformers **I** and **I^ROT^**, therefore, the stereoelectronic conditions are set to avoid decarboxylation of the β‐keto acid species. The equatorial carboxylate group brings the Cα−CO_2_^−^ bond just about in plane with the C=O bond of the ketone. Orbital overlap is, however, optimal for decarboxylation when the two bonds are roughly orthogonal, as would be the case when the carboxylate group is oriented axially.^[6]^ To compensate for the loss of stereoelectronic control in conformers featuring distortion of the sugar‐ring pucker, the enzyme might rely on differential hydrogen bonding to keep its tight “grip” on the axial carboxylate group. With Y149 oriented away from the 4‐keto group and directed towards the carboxylate group, electron release into the C5 and the O4 atoms is mitigated and the readiness for decarboxylation to occur quenched effectively. The **I^ROT^** conformer involves analogous interactions relevant for catalysis as for **I** and appears to be fully poised for reduction of the 4‐keto group, to complete the C4 epimerization without even a trace of decarboxylation. The final reduction step was, therefore, not analyzed.

Based on the computational evidence, we applied mutagenesis (see Supporting Information methods) to probe the binding pocket interactions of the carboxylate group of the 4‐keto intermediate in **I** and **I^ROT^**. In **I** (Figure S6), the carboxylate is coordinated by T126, S127, S128, and T178 (main chain NH), while in **I^ROT^** (Figure S6), it is coordinated by R185. Sequence comparison (Figure S7) shows that S128, T178, and R185 are unique to the UGAepi subclass of SDRs. Residues were individually substituted to remove the stabilizing interaction or to introduce a destabilizing one. In the case of R185, this gave rise to a series of variants thought to represent a graduated change from stabilization (wild type>R185K>R185H) to destabilization (R185D>R185A). Purified enzymes (Figures S8 and S9) that were confirmed to have incorporated NAD^+^ in the folded protein were assessed in UDP‐GlcA reactions (1.0 mM) by HPLC (Figures S10–S13) and NMR analysis. Table 1 summarizes the results. Full time courses are shown in Figures S14–S22.

**Table 1:** Activities and product ratios of BcUGAepi variants reacted with UDP‐GlcA (**1**) and UDP‐GalA (**2**). The activities were determined from the linear part of the time course, excluding the initial burst present in some reactions (Figures S14–S22 and S28–S30). The slope of the linear regression (mM min^−1^) was divided by the enzyme concentration (mg mL^−1^) to give the initial rate in μmol(min mg)^−1^, which equates to U mg^−1^.

Enzyme	Substrate	Activity [mU mg^−1^]	UDP‐GlcA (**1**, %)	UDP‐GalA (**2**, %)	UDP‐xylose (**3**, %)	UDP‐4‐ketopentose (**4**, %)	Burst^[e]^
Wild type	**1**	500	33	67	0	0	–
T126A	**1**	0.08	33^[a]^	67^[a]^	0^[a]^	0^[a]^	–
S127A	**1**	24.3	33	67	0	0	–
S128A	**1**	11.8	33	67	0	0	–
S128E	**1**	0.2	85.4^[b]^	12.1^[b]^	1.7^[b]^	0.8^[b]^	–
T178A	**1**	0.09	33^[a]^	67^[a]^	0^[a]^	0^[a]^	–
R185A	**1**	0.05	90.7^[c]^	0^[c]^	0^[c]^	9.3^[c]^	0.10
R185D	**1**	0.05 (≈2.6)	92.5^[c]^	0^[c]^	5.1^[c]^ (≈95)	2.4^[c]^ (≈5)	0.22
R185H	**1**	0.05 (≈5)	81.3^c]^	8.1^[c]^	9.0^[c]^ (≈95)	1.6^[c]^ (≈5)	2.43
R185K	**1**	0.3 (≈66)	51.1^[c]^	21.8^[c]^	26.8^[c]^ (≈98)	0.3^[c]^ (≈2)	–
Wild type	**2**	500	33	67	0	0	–
T126A	**2**	0.09	24.8	74.8	0	0.4	–
S127A	**2**	218	29.1	70.5	0.4^[d]^	0	–
R185H	**2**	0.1	18.3	70.7	5.0^[d]^	6.0	–

Unless stated otherwise, the ratios are reported after 24 h of reaction time. For R185H, R185D, and R185K enzymes, the activity and composition of the initial product burst (at 1 min) are given in brackets. Each reaction was performed at least in duplicate and the data accuracy is ±5 %. [a] Product ratios are after 48 h reaction. [b] Product ratios are after 2 h reaction. [c] Product ratios are after 2 h 30 min reaction. [d] Product is UDP‐pentose (UDP‐xylose and/or UDP‐l‐arabinose). [e] Total molarity of product burst after 1 min (μM) divided by the enzyme molarity (μM). Burst refers here to a specific time of sampling to capture a fast release of an initial product. This sampling may not have allowed analysis of the first enzyme turnover, however (see the entry for the R185H variant). Note: specific activities were determined here in lieu of a full set of kinetic parameters (*k*_cat_, *K*_m_) given the focus on the analysis of the product formation.

The specific activity was decreased by ≥10^3^‐fold in all variants, except in S127A and S128A that were, respectively, approximately 25‐ and 50‐fold less active than the wild type. The products formed depended on whether the mutation had targeted interactions with the **I** or the **I^ROT^** conformer. Variants targeting the **I** conformer converted UDP‐GlcA cleanly into UDP‐GalA, except for S128E which additionally showed a trace amount of decarboxylation (Figure S10). Strikingly, R185 variants, which affect the **I^ROT^** conformer, resulted predominantly in decarboxylation of about half (R185K, R185H) or all (R185A, R185D) of the UDP‐GlcA substrate (Table 1; Figure S10). The portion of substrate escaping decarboxylation during R185K and R185H reactions was converted with C4 stereoinversion into UDP‐GalA. The decarboxylated product was comprised of UDP‐4‐ketopentose and UDP‐xylose in relative amounts that varied depending on the enzymes (Table 1). R185A produced UDP‐4‐ketopentose only, while R185K produced UDP‐xylose with traces of UDP‐4‐ketopentose. R185H and R185D produced mixtures of UDP‐xylose/UDP‐4‐ketopentose in a ratio of 5.6 and 2.1, respectively. NMR data showed the identity of UDP‐xylose and ruled out UDP‐l‐arabinose (Figure S23). Reduction of the UDP‐4‐ketopentose was, therefore, stereospecific and retained the configuration at the C4‐position from the UDP‐GlcA substrate.

**Scheme 1:**
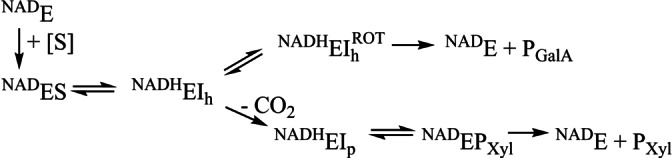
Proposed kinetic mechanism for the decarboxylation/epimerization reaction of R185 variants. E=enzyme; S=substrate; h=hexose; p=pentose.

The reaction of certain R185 variants (H, K, D) showed unusual kinetics: a fast initial release (referred to as “burst”) of decarboxylated products (UDP‐xylose, UDP‐4‐ketopentose) was followed by an approximately 10^2^‐fold slower formation of the product(s) with time (Table 1). Significantly, while the burst phase involved decarboxylation exclusively, the steady‐state reaction additionally gave the epimerized UDP‐GalA, in a relative portion of total product that was highly dependent on the enzyme used. From a series of kinetic experiments with the BcUGAepi (see Supporting Information methods; Figures S24 and S25), we identified tight binding/slow release of UDP‐xylose as the likely reason for the abrupt slowdown of the reaction at a high enzyme concentration. The burst could be eliminated by adding UDP‐xylose at the start of the reaction. A plausible kinetic mechanism for mixed decarboxylation‐epimerization by R185 variants is shown in Scheme 1.

The overall evidence from the enzyme variant study supports the idea that decarboxylation happens to the extent that site‐directed substitutions destabilize the **I^ROT^** conformer. Seeking additional support by independent methodology, we analyzed with MD simulation the **I^ROT^** conformers of all the R185 variants evaluated. The results explain the characteristic losses in activity and selectivity (epimerase compared to decarboxylase) in the variant enzymes arising from lowered precision in the positioning for catalysis and lack of conformational restraint for tight stereoelectronic control, respectively. Interatomic distances relevant for the immediate catalysis are found to be lengthened in the simulated **I^ROT^** conformers, especially of the low‐activity variants in comparison to the wild type (Figure S26a). Moreover, R185 variants, with the exception of R185K, involve extensive fluctuation between the ^4^ *C*_1_ and ^1^ *C*_4_ conformations of the sugar ring (Figure S26b–f), thus giving a structural rationale for decarboxylation as the principal reaction path of UDP‐GlcA conversion by these enzymes. In contrast, the change in the ^4^ *C*_1_ to ^1^ *C*_4_ conformation of the 4‐ketohexuronic acid, and the equatorial to axial reorientation of the carboxylate group associated with it, are not seen in MD simulations of the wild type **I^ROT^** conformer.

Lastly, from the evidence on the R185 variants, we examined the important suggestion that the decarboxylation happens to the extent that interactions between the binding pocket and the carboxylate group are disrupted in the opposite rotational isomer of the respective substrate used. Indeed, when offered UDP‐GalA as the substrate, variants affecting the **I** conformer (T126A, S127A) showed a small amount of decarboxylation (Table 1; Figure S27), clearly absent from their reactions with UDP‐GlcA. In contrast, R185H resulted in an approximately twofold smaller amount of decarboxylation and released relatively (ca. twofold) more epimerized product in the reaction with UDP‐GalA as compared to the reaction with UDP‐GlcA (Table 1; Figure S24). Full time courses are provided in the Supporting Information (Figures S28–S30).

## Conclusion

**Figure 4:**
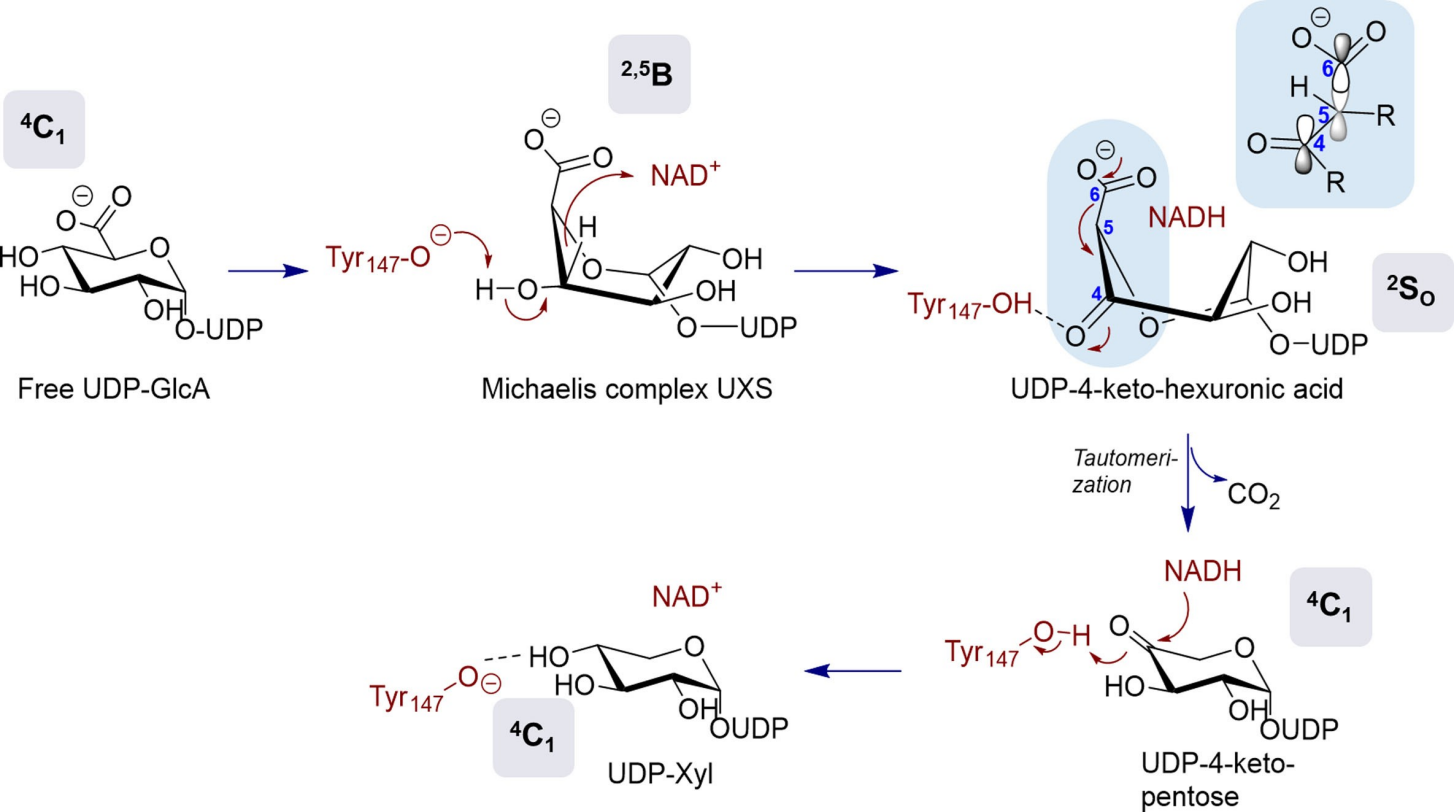
Proposed mechanism of UXS reacting with UDP‐GlcA and yielding UDP‐Xyl. The change in the ring pucker from the initial ^4^ *C*_1_ chair conformation in UDP‐GlcA to a ^2^ *S*_O_ skew‐boat conformation in the UDP‐4‐ketohexuronic acid intermediate brings the carboxylate moiety in an axial orientation, thus resulting in an optimal orbital alignment for rapid decarboxylation.[^6^, ^22^] See Figure 1 for comparison with UGAepi, which uses stereoelectronic control to prevent the decarboxylation.

In summary, our combined computational–experimental study reveals the coupled motions (coordinated conformational changes) used by BcUGAepi to reposition its UDP‐4‐ketohexuronic acid reaction intermediate for selective epimerization. We uncovered the role of enzyme‐promoted sugar ring distortion to steer a complex rotational itinerary of the 4‐keto intermediate, which is profoundly different from a simple rigid‐body rotation. SDR epimerases that use hexose/pentose nucleotide substrates (e.g. UDP‐galactose 4‐epimerase, UDP‐*N*‐acetyl‐glucosamine 4‐epimerase, and others) and so involve 4‐keto intermediates chemically less vulnerable than that of UGAepi, are thought (based on the static evidence of crystal structures) to make do in their catalysis with just rigid‐body rotations (Figure S31).[^3g^, ^3h^, ^17^, ^18^, ^19^, ^20^] Our results suggest the UGAepi‐specific strategies used to prevent decarboxylation of the 4‐ketohexuronic acid. First, the rotational endpoints **I** and **I^ROT^** manifest conformational sampling by the enzyme to implement stereoelectronic control. Second, dynamic rearrangement of the hydrogen bonding with the β‐keto acid moiety of the intermediate during the coupled motion can quench the reactivity of short‐lived sugar conformers that might otherwise decarboxylate easily. The UGAepi approach to handling the UDP‐4‐ketohexuronic acid has received significant mechanistic interest beyond epimerization, as a result of the existence of a distinct SDR family of decarboxylase enzymes.^[16]^ The reaction of these decarboxylases, exemplified by UDP‐xylose synthase (EC 4.1.1.35), involves the very same UDP‐4‐ketohexuronic acid species formed from enzyme‐NAD^+^ as in the UGAepi reaction, but proceeds from the 4‐keto intermediate exclusively through decarboxylation (Figure 4).[^3e^, ^21^, ^22^]

Evidence for distortion of the sugar ring (^4^ *C*_1_ → ^2,5^ *B* and ^2^ *S*_O_) concomitant with a change of the carboxylate group from an equatorial to an axial position in the UXS‐bound UDP‐GlcA are consistent with the stereoelectronic control from the enzyme now deployed to promote the decarboxylation optimally (Figure 4).^[22]^ Remarkably, therefore, site‐specific substitutions that interfere with the precise conformational sampling in BcUGAepi can convert the original epimerase into primitive UDP‐xylose synthases that decarboxylate UDP‐GlcA in slow, yet completely stereospecific reactions. Our findings thus connect protein conformational plasticity to enzymatic reactivity in UDP‐GlcA conversion. Given the diversity of transformations of sugar nucleotide substrates catalyzed by SDR enzymes,[^10^, ^11^, ^12^, ^15^, ^16^, ^23^] the evidence for a role of protein dynamics in the acquisition of a specific enzyme function can have broad relevance in a superfamily‐wide context. It can have practical relevance in ongoing efforts of enzyme discovery and engineering for the applied biocatalysis of sugar nucleotide synthesis.[^14^, ^24^] It is of mechanistic importance in linking conformational flexibility to reaction path selection and control. It contributes to an important field of current enzyme research at the crossroads of evolution, engineering and design.[^2f^, ^3a^, ^25^, ^26^]

## Experimental Section

Full details of the experimental and computational methods used are provided in the Supporting Information.

## Conflict of interest

The authors declare no conflict of interest.

1

## Supporting information

As a service to our authors and readers, this journal provides supporting information supplied by the authors. Such materials are peer reviewed and may be re‐organized for online delivery, but are not copy‐edited or typeset. Technical support issues arising from supporting information (other than missing files) should be addressed to the authors.

Supporting Information

Supporting Information

## Data Availability

The data that support the findings of this study are available from the corresponding authors upon reasonable request.
